# Comparable Outcomes in Redo Total Arch Replacement for Previous Aortic Dissection vs. Other Cardiac Surgeries: A Single-Center Pilot Study of the E-Vita Open Hybrid Prosthesis

**DOI:** 10.3390/jcm14217588

**Published:** 2025-10-26

**Authors:** Medhat Radwan, Luise Vöhringer, Michael Baumgaertner, Christoph Salewski, Spiros Lukas Marinos, Christian Jörg Rustenbach, Christian Schlensak, Isabelle Doll

**Affiliations:** 1 Department of Thoracic and Cardiovascular Surgery, Tübingen University Hospital, 72076 Tübingen, Germany; medhat.radwan@med.uni-tuebingen.de (M.R.); michael.baumgaertner@med.uni-tuebingen.de (M.B.); christoph.salewski@med.uni-tuebingen.de (C.S.); spiros.marinos@med.uni-tuebingen.de (S.L.M.); christian.rustenbach@med.uni-tuebingen.de (C.J.R.); christian.schlensak@med.uni-tuebingen.de (C.S.); 2 Department of Cardiovascular Surgery, Basel University Hospital, 4031 Basel, Switzerland; luise.voehringer@usb.ch

**Keywords:** frozen elephant trunk, aortic arch replacement, total arch replacement, aortic dissection, aortic aneurysm, E-vita open

## Abstract

**Background/Objectives:** Total arch replacement (TAR) with frozen elephant trunk (FET) using the E-vita Open hybrid prosthesis represents a complex surgical intervention for extensive aortic pathologies in previously operated patients. The comparative safety profile between patients with previous acute Type A dissection repair versus other cardiac surgical histories remains unclear. This pilot study evaluated early and midterm outcomes to determine whether previous aortic dissection carries additional operative risk compared to other previous cardiac operations. **Methods:** This retrospective single-center pilot cohort study analyzed 27 patients who underwent TAR with E-vita Open hybrid prosthesis between January 2013 and June 2024. Patients were stratified into two groups: Group 1 comprised patients with previous acute Type A dissection repair (*n* = 15, 55.6%), and Group 2 included patients with other previous cardiac operations (*n* = 12, 44.4%). Primary endpoints were in-hospital mortality and survival at 1, 2, and 3 years. Secondary endpoints included major neurological complications, spinal cord injury, reoperation for bleeding, and freedom from aortic reinterventions. **Results:** Baseline characteristics demonstrated comparable risk profiles between groups, with similar EuroSCORE II values (median 4.55 [IQR 3.86–7.28] vs. 5.41 [IQR 3.93–6.74], *p* = 1.0). Despite Group 1 showing trends toward longer operative times (580.07 ± 126.84 vs. 481.25 ± 119.29 min, *p* = 0.053), major postoperative outcomes were statistically equivalent. In-hospital mortality was 6.7% in Group 1 versus 0% in Group 2 (*p* = 1.0). Stroke rates were comparable (20.0% vs. 25.0%, *p* = 1.0), as were paraplegia rates (13.3% vs. 8.3%, *p* = 1.0) and dialysis requirements (46.7% vs. 41.7%, *p* = 0.334). Survival rates at 1, 2, and 3 years were 80.0%, 66.7%, and 60.0% for Group 1 and 75.0%, 66.7%, and 50.0% for Group 2, respectively (all *p* > 0.05). **Conclusions:** This pilot study suggests preliminary evidence of comparable early and midterm outcomes between patients with previous Type A dissection repair and those with other previous cardiac operations when undergoing TAR with E-vita Open hybrid prosthesis at an experienced center. However, the small sample size limits definitive conclusions and highlights the need for larger multicenter studies to confirm these findings.

## 1. Introduction

The surgical management of complex aortic arch pathology remains one of the most technically demanding challenges in cardiovascular surgery, with outcomes heavily dependent on institutional experience, patient selection, and technical expertise [1]. The introduction of hybrid techniques, particularly the frozen elephant trunk (FET) approach using the E-vita Open system, has fundamentally transformed the therapeutic landscape for extensive aortic disease, offering the potential for single-stage treatment of complex pathology extending from the aortic root to the descending thoracic aorta [2].

Recent international consensus statements have emphasized the importance of standardized approaches to FET procedures, particularly in complex patient populations [1,3,4].

This shift from traditional multi-stage procedures to single-stage hybrid interventions has been particularly significant for patients requiring reoperation, where the cumulative risks of multiple procedures must be carefully balanced against the technical complexity and potential morbidity of comprehensive single-stage repair [3]. Among the most challenging patient populations are those requiring reoperation following previous cardiac interventions. The technical complexity of redo sternotomy, combined with altered anatomy, extensive adhesions, and cumulative physiological stress, has traditionally been associated with increased perioperative risk and higher rates of major complications [5].

However, a critical knowledge gap exists regarding the specific impact of different types of previous cardiac operations on outcomes following total arch replacement (TAR) with FET, particularly comparing patients with previous acute Type A dissection repair versus other cardiac surgical histories [6,7,8]. Patients with previous acute Type A dissection repair present unique challenges that may influence subsequent surgical outcomes. The persistent presence of dissection membranes alters flow dynamics and causes progressive aneurysmal dilatation and potential false lumen patency, which creates a complex substrate for reoperation that may increase technical difficulty and complication rates [9].

Contemporary practice has increasingly favored conservative initial approaches for acute Type A dissection, with many centers adopting strategies of ascending aortic replacement with or without partial arch reconstruction during the acute phase, followed by staged completion of arch repair in survivors who develop progressive pathology [10]. This staged approach is predicated on the assumption that initial conservative management optimizes immediate survival during the acute phase, when patients are often critically ill with multiple organ dysfunction, while deferring more extensive reconstruction to a time when patients have recovered and can better tolerate complex procedures.

However, the validity of this staged approach critically depends on whether subsequent arch reconstruction in dissection survivors entails comparable risk to similar procedures in patients with other surgical histories—an area where current evidence remains limited. Therefore, this exploratory pilot study analyzes our institutional experience with total arch replacement using the E-vita Open hybrid prosthesis in previously operated patients, comparing early and midterm outcomes between those with prior acute Type A dissection repair and those with other cardiac surgical histories. We hypothesized that, in selected high-risk patients, redo FET surgery yields comparable outcomes and may thus represent a feasible treatment option.

## 2. Materials and Methods

### 2.1. Ethical Statement

Approval for this retrospective study was obtained from the local institutional review board and ethics committee of the University of Tübingen (Project number 005/2025BO2) on 15 January 2025. Due to the retrospective nature of this study, written informed consent was waived. All patient data were anonymized prior to analysis, and the study was conducted in accordance with the Declaration of Helsinki and local institutional guidelines for retrospective research.

### 2.2. Study Design and Patient Population

This retrospective single-center pilot analysis was conducted at the University Hospital Tübingen, Germany. From January 2013 to June 2024, patients undergoing thoracic aortic surgery were systematically screened using our institutional Aortic Registry. Fifty-one individuals who underwent TAR with FET technique using an E-vita Open stent graft (JOTEC/Artivion, Hechingen, Germany) were identified.

Inclusion criteria included age ≥ 18 years, previous cardiac surgery with documented operative reports, TAR with FET using E-vita Open prosthesis, and availability of complete perioperative data. Patients were excluded if they had isolated ascending or descending aortic replacement, partial arch replacement (defined as replacement of less than the entire arch), underwent TAR without the FET technique, underwent procedures employing stent graft systems other than the E-vita Open or underwent emergency procedures requiring immediate intervention without time for adequate preoperative planning.

Twenty-seven patients had a documented history of previous cardiac surgery and underwent FET as a redo procedure. These patients were stratified into two groups according to their prior cardiac operation: Group 1 included patients with previous type A dissection repair involving ascending or hemiarch replacement, and Group 2 comprised patients with other cardiac surgical interventions including coronary artery bypass grafting, valve replacement, or other aortic procedures. All operations were performed by senior cardiac surgeons with extensive experience in complex aortic surgery and encompassed both elective and urgent interventions.

**Figure 1:**
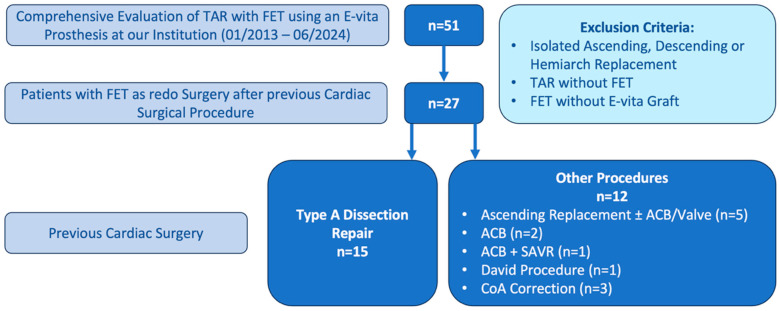
Patient Selection Process. TAR: Total Arch Replacement; FET: Frozen Elephant Trunk; ACB: Aorto-Coronary Bypass; SAVR: Surgical Aortic Valve Replacement; CoA: Coarctation of the Aorta.

The patient selection process is illustrated in Figure 1.

### 2.3. Power Analysis and Sample Size Justification

Given the retrospective nature of this study and the limited patient population available at our institution, no formal power calculation was performed prospectively. This exploratory pilot study design acknowledges the high risk for Type II error (failing to detect true differences between groups) due to small sample size and should be interpreted as hypothesis-generating research for larger multicenter investigations rather than definitive comparative effectiveness research.

### 2.4. Analyzed Parameters

Comprehensive perioperative information was retrospectively obtained from the institutional electronic medical records system and transferred into an Excel (Version 16.1, Microsoft, Redmond, WA, USA) database for analysis. To ensure reliability, data entry was double-checked by two independent researchers against the original electronic medical records. Preoperative variables included patient demographics, urgency of the procedure (elective, urgent, or emergent), comorbidities, relevant laboratory values, and operative risk assessment calculated using the EuroSCORE II.

Intraoperative parameters encompassed total procedure time, cardiopulmonary bypass (CPB) duration, aortic cross-clamp time, durations of selective cerebral perfusion and reperfusion, and information on any concomitant interventions. Data were collected on the specific E-vita prosthesis model implanted, cerebral perfusion strategy (unilateral, bilateral, or trilateral), and the Ishimaru classification of the distal anastomosis zone. Furthermore, nadir core temperature, peak lactate concentration, and the volumes or doses of administered blood products and hemostatic agents were systematically recorded.

### 2.5. Preoperative Management

The preoperative workup included comprehensive pulmonary function testing, laboratory analysis, computed tomography (CT) angiography, and transthoracic echocardiography. To mitigate the risk of spinal cord ischemia, elective patients routinely underwent preoperative cerebrospinal fluid (CSF) drainage, targeting a CSF pressure below 10 mmHg with a maximum drainage rate of 10 mL/h.

### 2.6. Surgical Technique

All procedures were performed via median sternotomy under general anesthesia with comprehensive monitoring. After systemic heparinization, CPB was established through right axillary artery cannulation using an 8-mm Dacron graft for antegrade selective cerebral perfusion (ASCP) and bicaval venous drainage.

Systemic cooling to a bladder temperature of 25–28 °C was routinely applied, corresponding to nasopharyngeal temperatures approximately 2–4 °C lower to ensure adequate neuroprotection. When circulatory arrest times > 60 min were anticipated, deeper cooling to 22–25 °C was used. Myocardial protection was achieved with intermittent cold blood cardioplegia. Circulatory arrest was followed by ASCP, with continuous cerebral oxygenation monitoring via bifrontal near-infrared spectroscopy (NIRS; Medtronic/Covidien INVOS™, Minneapolis, MN, USA and Masimo O3® Regional oximetry, Irvine, CA, USA).

The aortic arch was then excised, the true lumen of the descending thoracic aorta identified, and the selected E-vita prosthesis (Open, Open Plus, or Open Neo) deployed under direct vision with femoral guidewire assistance.

In chronic dissection, additional technical measures were applied to address anatomical complexity: true lumen identification by intraoperative transesophageal echocardiography and direct visualization after femoral guidewire placement, gentle balloon sizing when needed, and prosthesis sizing according to true lumen rather than total aortic diameter.

After systemic rewarming, the heart was de-aired, reperfused, and weaned from CPB under a target mean arterial pressure ≥ 70 mmHg. Patients were then transferred to the intensive care unit (ICU) for standardized postoperative management, including neurological monitoring, daily serum creatinine assessment, and comprehensive hemodynamic and organ surveillance.

### 2.7. Study Endpoints and Follow-Up

The primary study endpoint was all-cause in-hospital mortality. Secondary endpoints included perioperative stroke, spinal cord injury, reoperation for bleeding, acute kidney injury requiring renal replacement therapy, and other major complications. Midterm outcomes comprised survival at one, two, and three years, as well as the need for secondary thoracic or branched endovascular aortic repair (TEVAR/BEVAR). Stroke was defined as a clinically evident neurological deficit persisting > 24 h, corroborated by cerebral imaging (CT or magnetic resonance imaging (MRI)), and classified as either ischemic or hemorrhagic. Detailed assessment included timing of presentation (intraoperative, early postoperative < 48 h, or late postoperative >48h), severity assessment using National Institutes of Health Stroke Scale when available, and recovery status at discharge. Spinal cord injury was diagnosed based on clinical examination findings of motor or sensory deficits below the level of the lesion and confirmed by MRI when clinically indicated. In the event of postoperative motor deficits, CSF was released through the CSF drainage system to lower intrathecal pressure and improve spinal cord perfusion. Detection protocols included immediate postoperative neurological assessment and daily monitoring during ICU stay.

Data on survival and reinterventions were obtained through systematic outpatient clinic visits and structured telephone interviews. Contrast-enhanced CT imaging was performed at discharge, at three and six months, and annually thereafter as part of the routine follow-up protocol to assess graft integrity and detect aortic complications.

### 2.8. Statistical Analysis

All statistical analyses were conducted using IBM SPSS Statistics for Windows, Version 30.0 (IBM Corp., Armonk, NY, USA). Missing data were handled by case-wise deletion. No specific outlier treatment was applied, as all data points were considered to represent clinically plausible values. Continuous variables were first assessed for normality using the Shapiro–Wilk test. Normally distributed variables were expressed as mean ± standard deviation (SD) and compared between the two independent groups using the independent samples *t*-test. Non-normally distributed variables were reported as median and interquartile range (IQR) and compared using the Mann–Whitney U test.

Categorical variables were summarized as absolute frequencies and percentages. Group differences in categorical variables were analyzed using Chi-square tests. Where expected cell frequencies were less than 5 in more than 20% of cells, Fisher’s exact test was applied. All tests were two-sided, and a *p*-value of <0.05 was considered statistically significant.

## 3. Results

### 3.1. Baseline Characteristics and Risk Assessment

Baseline demographic and clinical characteristics are detailed in Table 1. As expected, underlying aortic pathology differed significantly between the groups, consistent with the study design. All other variables, including age, sex, anthropometric measures, comorbidities, preoperative risk scores, and the type and urgency of surgery, were comparable between groups.

Most procedures were performed electively (93.3% vs. 91.7%, *p* = 1.0). The median EuroSCORE II was 4.55 (IQR 3.86–7.28) in Group 1 and 5.41 (IQR 3.93–6.74) in Group 2 (*p* = 1.0), suggesting comparable predicted operative risk between groups. However, the EuroSCORE II may not fully capture the additional complexity associated with previous dissection repair.

### 3.2. Intraoperative Parameters and Technical Considerations

Intraoperative data are summarized in Table 2. CSF drainage was used in 80.0% of Group 1 patients and 75.0% of Group 2 patients (*p* = 1.0). The groups did not differ significantly with respect to total operative time, CPB duration, aortic cross-clamp time, reperfusion time, circulatory arrest time, cerebral perfusion time, nadir temperature, perfusion strategy, prosthesis type, Ishimaru zone of the distal anastomosis, arch vessel reimplantation pattern, concomitant procedures, or peak lactate concentration. Zone 0 distal anastomoses were performed in cases requiring extensive proximal aortic replacement extending to the sinotubular junction due to extensive disease involvement, not as part of debranching procedures.

However, Group 1 patients showed a trend toward longer operative times (580.07 ± 126.84 vs. 481.25 ± 119.29 min, *p* = 0.053), which may reflect the increased technical complexity of operating on previously dissected tissue. Nevertheless, other key intraoperative parameters were similar: cardiopulmonary bypass duration (254.07 ± 60.64 vs. 234.92 ± 53.01 min, *p* = 0.397), aortic cross-clamp time (110.80 ± 35.31 vs. 115.08 ± 43.09 min, *p* = 0.779), and circulatory arrest time (85.38 ± 25.22 vs. 64.75 ± 49.06 min, *p* = 0.308). Unilateral cerebral perfusion was used more frequently than bilateral perfusion in both groups (66.7% in both groups). E-vita Open was the most used prosthesis (60.0% vs. 50.0%).

### 3.3. Blood Product Utilization and Hemostatic Management

Table 3 outlines blood product utilization and coagulation factor administration. For each product, both the total volume or dose and the proportion of patients receiving the product were analyzed. No statistically significant differences were identified for red blood cell transfusion, platelet concentrate, fresh frozen plasma, prothrombin complex concentrate, fibrinogen, recombinant activated factor VII, von Willebrand factor concentrate, or factor XIII. The comparable blood product utilization between groups suggests that previous dissection repair did not significantly increase bleeding risk or hemostatic challenges during reoperation.

### 3.4. Postoperative Outcomes and Complications

Postoperative outcomes and complications are presented in Table 4. There were no statistically significant differences between groups in length of ICU stay, time to hospital discharge, need for re-sternotomy, postoperative stroke, renal insufficiency, dialysis requirement, delirium, multiorgan failure, paraplegia, short- and long-term survival, in-hospital mortality, or the requirement for subsequent endovascular reintervention.

The overall complication rates were notable, with stroke occurring in 20.0% of Group 1 patients and 25.0% of Group 2 patients (*p* = 1.0). Paraplegia occurred in 13.3% of Group 1 patients and 8.3% of Group 2 patients (*p* = 1.0). Survival rates at 1, 2, and 3 years were 80.0%, 66.7%, and 60.0% for Group 1, and 75.0%, 66.7%, and 50.0% for Group 2, respectively. Freedom from aortic reintervention was comparable between groups, with subsequent endovascular interventions required in 53.3% of Group 1 patients and 50.0% of Group 2 patients (*p* = 0.678).

## 4. Discussion

This pilot study directly addresses the clinically important question of whether patients with previous Type A dissection repair face different risks when undergoing subsequent TAR with E-vita Open hybrid prosthesis compared to patients with other previous cardiac operations. While our findings suggest comparable early and midterm outcomes between groups, the small sample size and single-center experience limit our ability to make definitive conclusions, and these results should be considered hypothesis-generating for larger studies.

### 4.1. Principal Findings and Clinical Context

The comparable outcomes between groups, despite the theoretically increased complexity of operating on dissected aortic tissue, provide preliminary support for several important clinical observations. The 6.7% in-hospital mortality in the previous dissection group aligns reasonably well with contemporary reports from the International E-vita Open Registry, which documented an overall 30-day mortality of 12% across all patient populations [11].

Our lower mortality rate likely reflects careful patient selection, institutional experience accumulated over the 11-year study period, and the predominant elective nature of procedures (93.3%). The EuroSCORE II values were comparable between groups, suggesting similar predicted operative risk, though this scoring system may not fully capture the additional complexity associated with previous dissection repair, including technical challenges related to tissue planes, false lumen management, and altered anatomy.

### 4.2. Operative Outcomes and Technical Considerations

A key finding of this study is the trend toward longer operative times in patients with previous Type A dissection repair compared to those with other prior cardiac surgeries, although this difference did not reach statistical significance (580.07 ± 126.84 vs. 481.25 ± 119.29 min, *p* = 0.053). This observation aligns with the widely accepted notion that reoperations in the context of previous dissection are technically more demanding. The dissected tissues are often fragile, and the presence of a chronic dissection flap can obscure anatomical planes, making the identification and mobilization of structures more challenging and time-consuming [5]. Furthermore, the need for meticulous management of the false lumen to prevent pressurization and ensure adequate distal perfusion adds another layer of complexity to the procedure [9].

Despite the increased technical challenges, it is noteworthy that other intraoperative parameters, such as CPB duration, aortic cross-clamp time, and circulatory arrest time, were comparable between the two groups. This suggests that while the overall procedure may be longer, the core components of the operation can be performed with similar efficiency in both patient populations.

Our institutional approach to these technical challenges evolved during the study period. Early cases required longer operative times as we refined techniques for true lumen identification and appropriate sizing in dissected anatomy. The implementation of standardized protocols for chronic dissection management, including routine use of balloon sizing and acceptance of partial false lumen exclusion, contributed to more predictable operative times in later cases.

The comparable blood product utilization between the groups further supports this, indicating that the increased technical complexity did not translate into an increased bleeding risk or hemostatic challenges during reoperation. This is reassuring given theoretical concerns about tissue fragility and altered coagulation in reoperative settings and likely reflects both a standardized surgical strategy and the experience of the operating team in managing these complex cases.

Our findings are consistent with other studies that have reported on redo aortic arch surgery after previous Type A dissection repair. For instance, a study by Ohira et al. revealed that reoperative aortic arch repair after Type A dissection repair is safe and durable in experienced centers [12]. Similarly, Bajona et al. reported that reoperation after acute dissection repair can be accomplished safely, with acceptable early and late outcomes [13]. These studies, along with our own, support the concept of a staged approach to the management of complex aortic dissections, where an initial, less extensive repair is performed in the acute setting, followed by a definitive, more extensive repair in the elective setting.

### 4.3. Neurological Outcomes and Protection Strategies

Stroke rates of 20.0% in the dissection group and 25.0% in the control group, while statistically comparable between groups, are concerning and exceed those reported in some contemporary series. Cefarelli and colleagues, in their comprehensive analysis of 791 patients undergoing elective aortic arch repair, reported overall stroke rates of 3.9% in elective procedures, though this series excluded the reoperative complexity inherent in our cohort [14]. A multicenter study evaluating FET as redo surgery following ascending aortic replacement for Type A dissection reported a postoperative stroke rate of 15%. Notably, their patient cohort had a higher baseline prevalence of preoperative stroke compared with ours (22% vs. 13.3%) [6]. Recent reviews have highlighted ongoing refinements in cerebral protection strategies, including the shift toward moderate hypothermia combined with ASCP to minimize neurological injury [15,16]. Our institutional approach follows these principles, although stroke rates in our cohort remain higher than reported in some contemporary series.

The elevated stroke rates in our series may reflect several factors, including the complexity of reoperative procedures, the learning curve associated with FET techniques, and potential selection bias toward higher-risk patients. The comparable rates between groups suggest that previous dissection repair per se may not increase neurological risk, but the overall rates are concerning and highlight the need for continued refinement of cerebral protection strategies. These findings underscore the importance of careful patient selection and the need for centers performing these procedures to maintain rigorous outcome monitoring and quality improvement programs.

The paraplegia rates of 13.3% and 8.3%, respectively, though not statistically different between groups, represent one of the most devastating complications of FET procedures and highlight the ongoing challenge of spinal cord protection in complex aortic surgery. In addition to the stroke outcomes discussed above, the same multicenter study reported a postoperative paraplegia rate of 3%, which is considerably lower than in our cohort, reflecting the high-risk nature of our patient population [6]. Recent advances in spinal cord protection strategies, including prophylactic CSF drainage, comprehensive neuroprotection protocols, and staged procedures to minimize ischemic burden, have shown promise in reducing these complications [17].

Murana and colleagues demonstrated that preventive measures, particularly CSF drainage with peridural pressure monitoring, can significantly reduce the occurrence of spinal cord injuries in extended aortic coverage procedures [18]. The implementation of such protocols in our institution during the latter part of the study period may have contributed to the acceptable neurological outcomes, though the small sample size precludes formal analysis of temporal trends in complication rates.

The neurological complications observed in this series highlight the critical importance of comprehensive neuroprotection strategies in complex aortic arch surgery. These should include optimized cerebral perfusion techniques, careful temperature management, appropriate use of CSF drainage, and consideration of staged procedures in high-risk patients. The development of institutional protocols for neurological monitoring and intervention may be crucial for improving outcomes in this challenging patient population.

### 4.4. Survival and Long-Term Outcomes

The three-year survival rates (60.0% vs. 50.0%) reflect the complex nature of patients undergoing redo arch operations, many with multiple comorbidities and advanced aortic disease.

These preliminary results provide some support for staged surgical approaches to acute Type A dissection management, where initial conservative management with ascending aortic or hemiarch replacement during the acute phase is followed by elective arch completion in survivors who develop progressive pathology [19]. This staged approach potentially optimizes both immediate survival during the acute phase, when patients are often critically ill with multiple organ dysfunction, and long-term aortic management outcomes by allowing for patient stabilization and careful planning of definitive reconstruction.

Extensive literature across multiple surgical specialties, including aortic surgery, consistently demonstrates superior outcomes at high-volume centers with experienced teams [20]. This volume-outcome relationship is particularly relevant for complex procedures such as TAR with FET, where technical expertise, institutional protocols, and multidisciplinary team coordination are crucial for optimal outcomes.

The high rate of subsequent endovascular interventions (53.3% vs. 50.0%) reflects the complex nature of aortic disease in these patients and the complementary role of endovascular techniques in modern aortic management. This finding supports the concept of hybrid aortic management, where surgical and endovascular techniques are used in combination to achieve optimal long-term outcomes. The comparable rates between groups suggest that the need for subsequent interventions is related to the underlying aortic pathology rather than the specific type of previous cardiac surgery.

### 4.5. Study Limitations and Methodological Considerations

This study has several important limitations that must be acknowledged and considered when interpreting the results. The most significant limitation is the small sample size, which severely limits statistical power and increases the risk of Type II error (failing to detect true differences between groups). With only 27 patients divided into two groups, the study is underpowered to detect clinically meaningful differences in outcomes, particularly for rare events such as mortality and major complications.

The retrospective design introduces several potential biases, including selection bias in patient choice for surgery, information bias in data collection, and survival bias (only patients surviving to reoperation are included). The single-center experience, while ensuring consistency in surgical technique and perioperative management, limits generalizability to centers with different experience levels, patient populations, or practice patterns.

The heterogeneous nature of previous cardiac operations in the control group introduces potential confounding variables that may influence outcomes. Patients with previous coronary artery bypass grafting may have different risk profiles compared to those with previous valve surgery, and these differences could impact outcomes independent of the specific comparison of interest. A more homogeneous control group would strengthen the validity of the comparison. Furthermore, quality of life assessments is beyond our retrospective analysis and detailed cost-effectiveness analyses would provide valuable insights for clinical decision-making.

## 5. Conclusions

This study provides preliminary evidence that patients with previous Type A dissection repairs may achieve comparable early and midterm outcomes to those with other previous cardiac operations when undergoing TAR with E-vita Open hybrid prosthesis at an experienced center. The high rates of neurological complications observed in both groups underscore the significant morbidity associated with these procedures and the importance of careful patient selection and meticulous surgical technique. However, the small sample size limits definitive conclusions, and these findings should be considered hypothesis-generating for larger multicenter investigations.

## Figures and Tables

**Table 1:** Baseline characteristics.

Variable	Previous Type A Dissection Repair (*n* = 15)	Previous Other Cardiac Surgery (*n* = 12)	*p*-Value
Age [years]	59.80 ± 7.94	61.83 ± 14.54	0.669 ^TT^
Gender [male]	10 (66.7%)	4 (33.3%)	0.085 ^Chi2^
Height [cm]	173.53 ± 12.35	169.83 ± 18.01	0.533 ^TT^
Weight [kg]	88.00 ± 18.83	84.67 ± 18.65	0.65 ^TT^
Diabetes			0.767 ^Fisher^
OAD	0 (0.0%)	1 (8.3%)	
Insulin dependent	0 (0.0%)	0 (0.0%)	
Unmedicated	2 (13.3%)	1 (8.3%)	
Hypertension	15 (100.0%)	12 (100.0%)	n.a.
Hyperlipidemia	10 (66.7%)	9 (75.0%)	0.696 ^Fisher^
COPD	1 (6.7%)	0 (0.0%)	1.0 ^Fisher^
Smoking history	5 (33.3%)	3 (25.0%)	0.696 ^Fisher^
Stroke	2 (13.3%)	0 (0.0%)	0.487 ^Fisher^
Renal insufficiency			0.342 ^Fisher^
0: none	2 (13.3%)	3 (25.0%)	
1: GFR > 89 [mL/min]	0 (0.0%)	1 (8.3%)	
2: GFR 60–89 [mL/min]	8 (53.3%)	7 (58.3%)	
3: GFR 30–59 [mL/min]	5 (33.3%)	1 (8.3%)	
4: GFR 15–29 [mL/min]	0 (0.0%)	0 (0.0%)	
5: GFR < 15 [mL/min]	0 (0.0%)	0 (0.0%)	
Dialysis	0 (0.0%)	0 (0.0%)	
EuroSCORE II	4.55 (3.86–7.28)	5.41 (3.93–6.74)	1.0 ^MW^
Aortic pathology			<0.001 ^Fisher^
Dissection	1 (6.7%)	1 (8.3%)	
Aneurysm	1 (6.7%)	9 (75.0%)	
Combination	13 (86.7%)	2 (16.7%)	
Type of surgery			1.0 ^Fisher^
Elective	14 (93.3%)	11 (91.7%)	
Urgent	1 (6.7%)	1 (8.3%)	
Emergent	0 (0.0%)	0 (0.0%)	
CS-Bypass before FET	6 (40.0%)	3 (25.0%)	0.683 ^Fisher^

Values shown as mean (±SD), median (IQR) or *n* (%). OAD: Oral Antidiabetic Drug; COPD: Chronic Obsturctive Pulmonary Disease; GFR: Glomerular Filtration Rate; CS-Bypass: Carotid-Subclavian Bypass; FET: Frozen Elephant Trunk; n.a.: not applicable. ^TT^: T-Test, ^Chi2^: Chi-Square-Test, ^Fisher^: Fisher’s Exact Test, ^MW^: Mann–Whitney U Test.

**Table 2:** Intraoperative parameters.

Variable	Previous Type A Dissection Repair (*n* = 15)	Previous Other Cardiac Surgery (*n* = 12)	*p*-Value
CSF drainage	12 (80%)	9 (75%)	1.0 ^Fisher^
Duration of surgery [min]	580.07 ± 126.84	481.25 ± 119.29	0.053 ^TT^
CPB time [min]	254.07 ± 60.64	234.92 ± 53.01	0.397 ^TT^
Cross-clamp time [min]	110.80 ± 35.31	115.08 ± 43.09	0.779 ^TT^
Reperfusion [min]	80.80 ± 35.66	75.50 ± 18.01	0.644 ^TT^
Circulatory arrest [min]	85.38 ± 25.22 (n = 8)	64.75 ± 49.06 (n = 8)	0.308 ^TT^
Cerebral perfusion [min]	89.60 ± 20.30	78.27 ± 38.70	0.391 ^TT^
Lowest Temperature [°C]	27.5 (26.0–28.5)	26.25 (25.83–28.0)	0.183 ^MW^
Perfusion Strategy			0.218 ^Fisher^
Unilateral	10 (66.7%)	8 (66.7%)	
Bilateral	3 (20.0%)	3 (25.0%)	
Trilateral	2 (13.3%)	4 (33.3%)	
Prosthesis type			0.87 ^Fisher^
Open	9 (60.0%)	6 (50.0%)	
Open Plus	2 (13.3%)	3 (25.0%)	
Open Neo	4 (26.7%)	3 (25.0%)	
Ishimaru zone of distal anastomosis			0.376 ^Fisher^
0	2 (13.3%)	3 (25.0%)	
2	7 (46.7%)	3 (25.0%)	
3	3 (20.0%)	5 (41.7%)	
Arch vessel reimplantation			0.13 ^Fisher^
Truncus, carotid, subclavia	4 (26.7%)	7 (58.3%)	
Truncus, carotid	11 (73.3%)	5 (41.7%)	
Concomitant procedure			0.332 ^Fisher^
ACB	0 (0.0%)	1 (8.3%)	
Valve	2 (13.3%)	0 (0.0%)	
Max. Lactate [mmol/L]	8.24 ± 2.55	7.04 ± 3.70	0.352 ^TT^

Values shown as mean (±SD), median (IQR) or n (%). CSF: Cerebrospinal Fluid. ^TT^: T-Test, ^Fisher^: Fisher’s Exact Test, ^MW^: Mann–Whitney U Test.

**Table 3:** Intraoperative transfusion of blood and coagulatory components.

Variable	Previous Type A Dissection Repair (*n* = 15)	Previous Other Cardiac Surgery (*n* = 12)	*p*-Value
RBC [mL]	1650 (825–3600) 14 (93.3%)	1350 (900–3975) 12 (100.0%)	0.899 ^MW^ 1.0 ^Fisher^
Platelets [mL]	1275 ± 773.29 14 (93.3%)	1425 ± 822.34 10 (83.3%)	0.653 ^TT^ 0.569 ^Fisher^
FFP [mL]	2760 ± 1347.59 10 (66.7%)	2416.67 ± 1942.59 6 (50.0%)	0.681 ^TT^ 0.452 ^Fisher^
PCC [I.U.]	6000 (4000–8000) 15 (100.0%)	6000 (4000–9000) 11 (91.7%)	0.919 ^MW^ 0.444 ^Fisher^
Fibrinogen [g]	6.0 (4.0–16.0) 15 (100.0%)	5.5 (4.0–7.3) 10 (83.3%)	0.428 ^MW^ 0.188 ^Fisher^
NovoSeven [mg]	8 (6.5–18) 5 (33.3%)	6.1 (4.2–n.a.) 2 (16.7%)	0.381 ^MW^ 0.408 ^Fisher^
Haemate [I.U.]	3500 (3000–5000) 8 (53.3%)	2000 (1250–3500) 4 (33.3%)	0.073 ^MW^ 0.441 ^Fisher^
Fibrogammin [I.U.]	1875 (1250–n.a.) 2 (13.3%)	1875 (1250–n.a.) 2 (16.7%)	1.0 ^MW^ 1.0 ^Fisher^

Values shown as mean (±SD), median (IQR) or n (%). RBC: Red Blood Cells; FFP: Fresh Frozen Plasma, PCC: Prothrombin Complex Concentrate, ^TT^: T-Test, ^Fisher^: Fisher’s Exact Test, ^MW^: Mann–Whitney U Test. n.a.: Not applicable. The 3rd quartile could not be estimated due to the small sample size.

**Table 4:** Postoperative cohort characteristics and complications.

Variable	Previous Type A Dissection Repair (*n* = 15)	Previous Other Cardiac Surgery (*n* = 12)	*p*-Value
LOS ICU [d]	10 (8–24)	6.5 (2.25–17.75)	0.114 ^MW^
Time to discharge [d]	24 (18–40.5)	22.5 (16.75–33.0)	0.631 ^MW^
Re-Sternotomy	4 (26.7%)	2 (16.7%)	0.662 ^Fisher^
Stroke	3 (20.0%)	3 (25.0%)	1.0 ^Fisher^
Renal insufficiency			0.334 ^Fisher^
0: none	0 (0.0%)	0 (0.0%)	
1: GFR > 89 [mL/min]	0 (0.0%)	0 (0.0%)	
2: GFR 60–89 [mL/min]	0 (0.0%)	3 (25.0%)	
3: GFR 30–59 [mL/min]	3 (20.0%)	3 (25.0%)	
4: GFR 15–29 [mL/min]	3 (20.0%)	1 (8.3%)	
5: GFR < 15 [mL/min]	1 (6.7%)	0 (0.0%)	
Dialysis	7 (46.7%)	5 (41.7%)	
Delirium	5 (33.3%)	3 (25.0%)	0.696 ^Fisher^
Multiorgan failure	1 (6.7%)	1 (8.3%)	1.0 ^Fisher^
Paraplegia	2 (13.3%)	1 (8.3%)	1.0 ^Fisher^
Survival			
30 d	14 (93.3%)	12 (100.0%)	1.0 ^Fisher^
1 y	12 (80.0%)	9 (75.0%)	0.502 ^Fisher^
2 y	10 (66.7%)	8 (66.7%)	0.495 ^Fisher^
3 y	9 (60.0%)	6 (50.0%)	1.0 ^Fisher^
In-Hospital Mortality	1 (6.7%)	0 (0.0%)	1.0 ^Fisher^
Subsequent endovascular procedure (TEVAR/BEVAR)	8 (53.3%)	6 (50.0%)	0.678 ^Fisher^

Values shown as median (IQR) or n (%). GFR: Glomerular Filtration Rate; TEVAR: Thoracic Endovascular Aortic Repair; BEVAR: Branched Endovascular Aortic Repair. ^Fisher^: Fisher’s Exact Test, ^MW^: Mann–Whitney U Test.

## Data Availability

The data underlying this article are available in the article.

## Abbreviations

The following abbreviations are used in this manuscript:

ACB	Aorto-Coronary Bypass
ASCP	Antegrade Selective Cerebral Perfusion
BEVAR	Branched Endovascular Aortic Repair
Chi2	Chi-Square-Test
CoA	Coarctation of the Aorta
COPD	Chronic Obstructive Pulmonary Disease
CPB	Cardiopulmonary Bypass
CS	Carotid-subclavian
CSF	Cerebrospinal Fluid
CT	Computed Tomography
FET	Frozen Elephant Trunk
FFP	Fresh Frozen Plasma
Fisher	Fisher’s Exact Test
GFR	Glomerular Filtration Rate
ICU	Intensive Care Unit
IQR	Interquartile Range
MRI	Magnetic Resonance Imaging
MW	Mann–Whitney U Test
n.a.	not applicable
NIRS	Near-Infrared Spectroscopy
OAD	Oral Antidiabetic Drugs
PCC	Prothrombin Complex Concentrate
RBC	Red Blood Cells
SAVR	Surgical Aortic Valve Replacement
SD	Standard Deviation
TAR	Total Arch Replacement
TEVAR	Thoracic Endovascular Aortic Repair
TT	T-Test

## References

[1] Czerny M., Schmidli J., Adler S., van den Berg J.C., Bertoglio L., Carrel T., Chiesa R., Clough R.E., Eberle B., Etz C. (2019). Current options and recommendations for the treatment of thoracic aortic pathologies involving the aortic arch: An expert consensus document of the European Association for Cardio-Thoracic surgery (EACTS) and the European Society for Vascular Surgery (ESVS). Eur. J. Cardiothorac. Surg..

[2] Song S.W., Lee H., Kim M.S., Wong R.H.L., Ho J.Y.K., Szeto W.Y., Jakob H. (2024). Next-Generation Frozen Elephant Trunk Technique in the Era of Precision Medicine. J. Chest Surg..

[3] Shrestha M., Bachet J., Bavaria J., Carrel T.P., De Paulis R., Di Bartolomeo R., Etz C.D., Grabenwöger M., Grimm M., Haverich A. (2015). Current status and recommendations for use of the frozen elephant trunk technique: A position paper by the Vascular Domain of EACTS. Eur. J. Cardio Thorac. Surg..

[4] Czerny M., Grabenwöger M., Berger T., Aboyans V., Della Corte A., Chen E.P., Desai N.D., Dumfarth J., Elefteriades J.A., Etz C.D. (2024). EACTS/STS Guidelines for Diagnosing and Treating Acute and Chronic Syndromes of the Aortic Organ. Ann. Thorac. Surg..

[5] Kozlov B.N., Panfilov D.S., Kim E.B. (2024). Long-term outcomes of frozen elephant trunk for aortic dissection: A single-center experience. J. Cardiothorac. Surg..

[6] Kreibich M., Pitts L., Kempfert J., Yildiz M., Schönhoff F., Gaisendrees C., Luehr M., Berger T., Demal T., Jahn J. (2024). Multicentre frozen elephant trunk technique experience as redo surgery to treat residual type A aortic dissections following ascending aortic replacement. Eur. J. Cardio Thorac. Surg..

[7] Dietze Z., Kang J., Madomegov K., Etz C.D., Misfeld M., Borger M.A., Leontyev S. (2023). Aortic arch redo surgery: Early and mid-term outcomes in 120 patients. Eur. J. Cardio Thorac. Surg..

[8] Demal T.J., Bax L., Brickwedel J., Kölbel T., Vettorazzi E., Sitzmann F., Reichenspurner H., Detter C. (2021). Outcome of the frozen elephant trunk procedure as a redo operation. Interact. Cardiovasc. Thorac. Surg..

[9] Tsagakis K., Tossios P., Kamler M., Benedik J., Natour D., Eggebrecht H., Piotrowski J., Jakob H. (2011). The DeBakey classification exactly reflects late outcome and re-intervention probability in acute aortic dissection with a slightly modified type II definition. Eur. J. Cardiothorac. Surg..

[10] Leone A., Beckmann E., Martens A., Di Marco L., Pantaleo A., Reggiani L.B., Haverich A., Di Bartolomeo R., Pacini D., Shrestha M. (2020). Total aortic arch replacement with frozen elephant trunk technique: Results from two European institutes. J. Thorac. Cardiovasc. Surg..

[11] Tsagakis K., Pacini D., Grabenwöger M., Borger M.A., Goebel N., Hemmer W., Laranjeira Santos A., Sioris T., Widenka K., Risteski P. (2020). Results of frozen elephant trunk from the international E-vita Open registry. Ann. Cardiothorac. Surg..

[12] Ohira S., Tavolacci S.C., de la Pena C., Spielvogel D. (2025). Outcomes of Redo Aortic Arch Repair Post Type A Dissection in the Modern Era. Seminars in Thoracic and Cardiovascular Surgery.

[13] Bajona P., Quintana E., Schaff H.V., Daly R.C., Dearani J.A., Greason K.L., Pochettino A. (2015). Aortic arch surgery after previous type A dissection repair: Results up to 5 years. Interact. Cardiovasc. Thorac. Surg..

[14] Cefarelli M., Murana G., Surace G.G., Castrovinci S., Jafrancesco G., Kelder J.C., Klein P., Sonker U., Morshuis W.J., Heijmen R.H. (2017). Elective Aortic Arch Repair: Factors Influencing Neurologic Outcome in 791 Patients. Ann. Thorac. Surg..

[15] Werner P., Winter M., Mahr S., Stelzmueller M.-E., Zimpfer D., Ehrlich M. (2024). Cerebral Protection Strategies in Aortic Arch Surgery—Past Developments, Current Evidence, and Future Innovation. Bioengineering.

[16] Myers A., Nita C., Martinez G. (2025). Cerebral and Spinal Cord Protection Strategies in Aortic Arch Surgery. J. Cardiovasc. Dev. Dis..

[17] Zhou C., Hou B., Zhang K., Gao S., Cao F., Ji Y., Xie E., Qiu J., Qiu J., Yu C. (2025). Protective Effect on Spinal Cord Injury of Prophylactic Cerebrospinal Fluid Drainage in Extensive Aortic Arch Repair for Type a Aortic Dissection: A Retrospective Cohort Study. J. Am. Heart Assoc..

[18] Murana G., Campanini F., Fiaschini C., Barberio G., Folesani G., Pacini D. (2023). Spinal cord injury after frozen elephant trunk procedures-prevention and management. Ann. Cardiothorac. Surg..

[19] Biancari F., Juvonen T., Fiore A., Perrotti A., Hervé A., Touma J., Pettinari M., Peterss S., Buech J., Dell’Aquila A.M. (2023). Current Outcome after Surgery for Type a Aortic Dissection. Ann. Surg..

[20] Holt P.J., Poloniecki J.D., Gerrard D., Loftus I.M., Thompson M.M. (2007). Meta-analysis and systematic review of the relationship between volume and outcome in abdominal aortic aneurysm surgery. Br. J. Surg..

